# Predictors of cochlear implant outcomes in pediatric auditory neuropathy: A matched case-control study

**DOI:** 10.1371/journal.pone.0304316

**Published:** 2024-05-29

**Authors:** Zahra Jafari, Elizabeth M. Fitzpatrick, David R. Schramm, Isabelle Rouillon, Amineh Koravand

**Affiliations:** 1 Audiology and Speech-Language Pathology Program, University of Ottawa, Ottawa, Ontario, Canada; 2 School of Communication Sciences and Disorders (SCSD), Dalhousie University, Halifax, NS, Canada; 3 Child Hearing Lab, CHEO Research Institute, University of Ottawa, Ottawa, Ontario, Canada; 4 Department of Otolaryngology-Head and Neck Surgery, University of Ottawa, Ottawa, Ontario, Canada; 5 Department of Otolaryngology-Head and Neck Surgery, The Ottawa Hospital, Ottawa, Ontario, Canada; 6 Speech and Language Pathology, and Otolaryngology Department, Necker Hospital, Paris, France; UFPE: Universidade Federal de Pernambuco, BRAZIL

## Abstract

**Objectives:**

Current evidence supports the benefits of cochlear implants (CIs) in children with hearing loss, including those with auditory neuropathy spectrum disorder (ANSD). However, there is limited evidence regarding factors that hold predictive value for intervention outcomes.

**Design:**

This retrospective case-control study consisted of 66 children with CIs, including 22 with ANSD and 44 with sensorineural hearing loss (SNHL) matched on sex, age, age at CI activation, and the length of follow-up with CIs (1:2 ratio). The case and control groups were compared in the results of five open-set speech perception tests, and a Forward Linear Regression Model was used to identify factors that can predict the post-CI outcomes.

**Results:**

There was no significant difference in average scores between the two groups across five outcome measures, ranging from 88.40% to 95.65%. The correlation matrix revealed that younger ages at hearing aid fitting and CI activation positively influenced improvements in speech perception test scores. Furthermore, among the variables incorporated in the regression model, the duration of follow-up with CIs, age at CI activation, and the utilization of two CIs demonstrated prognostic significance for improved post-CI speech perception outcomes.

**Conclusions:**

Children with ANSD can achieve similar open-set speech perception outcomes as children with SNHL. A longer CI follow-up, a lower age at CI activation, and the use of two CIs are predictive for optimal CI outcome.

## Introduction

Auditory neuropathy spectrum disorder (ANSD) is observed in 4% to 5% of all degrees of hearing loss, and 10% to 15% of school-age children with severe to profound sensorineural hearing loss (SNHL) [1, 2]. ANSD is characterized by hearing impairment despite normally functioning outer hair cells (OHCs) [3]. In clinical audiology, ANSD is diagnosed by present otoacoustic emissions (OAE) and/or cochlear microphonics (CM), concomitant with the abnormal transmission of auditory signals from cochlear synapses to the brain as evidenced by absent or severely abnormal auditory brainstem responses (ABRs) [3, 4]. The lesion site causing ANSD may vary from the presynaptic site of release of glutamate in the inner hair cells (IHCs) to the cochlear synapse, the postsynaptic site of neurotransmitter stimulation, the site of initiation of the excitatory postsynaptic potential (EPSP) at the terminal dendrite, or sites along the spiral ganglion, which affect auditory signal transmission along the auditory nerve to the brain [3, 4]. In ANSD, deficiency in neural transmission (due to a reduced number of activated auditory nerve fibers or deafferentation) and/or neural dyssynchrony (due to progressive demyelination) are the two basic mechanisms that contribute to the disruption of neural activity and temporal resolution deficits at the auditory brainstem level [5–7]. Individuals with ANSD typically show difficulty with the temporal processing of sound resulting in impaired speech perception, especially in background noise [8–10].

The etiology of ANSD is complex and can broadly be classified into acquired and genetic factors [11]. A wide range of acquired factors, such as perinatal conditions, neurometabolic diseases, immune disorders, and ototoxic drug exposure might contribute to ANSD [11–13]. More than 40% of patients with auditory neuropathy have a genetic predisposition [11], including autosomal recessive (e.g., OTOF, PJVK), autosomal dominant (e.g., OPA1, MPZ, ATP1A3, SLC17A8, DIAPH3), X-linked (e.g., AIFM1), and mitochondrial mutations during maternal inheritance [14, 15].

Intervention and management for ANSD are challenging and require a team approach including parental education about ANSD management [13, 16]. Hearing technology interventions for ANSD vary according to individual cases due to the extent of the lesion and disease severity [17]. The American Academy of Audiology (AAA) Pediatric Amplification Guidelines recommend a hearing aid (HA) trial for children with reliable, permanent HL that interferes with speech perception at conversational levels [18]. However, HAs may not be an appropriate option for many patients with ANSD as making sounds louder does not improve the processing of auditory temporal cues [19, 20]. Cochlear implantation has revolutionized the care for individuals with severe to profound hearing loss (HL). Given that the perception of auditory information is largely based on temporal processing, which can be successfully transmitted by only a few electrodes [21], cochlear implants (CIs) are the standard of care for many patients with ANSD [3]. The CI bypasses the lesion site (i.e., IHCs and cochlear synapses) and directly stimulates the spiral ganglion neurons. Further, compared to acoustic stimulation, electric stimulation is superior in stimulating and synchronizing auditory nerve fibers [3], which enhances neural synchrony and allows the development of fundamental speech and hearing skills [22].

Similar to children with SNHL [23], better outcomes in children with ANSD are associated with receiving hearing interventions (CIs or HAs) before three years of age [20, 24, 25]. While existing evidence demonstrates the benefits of CIs for both children with SNHL and ANSD [7, 17, 26–29], factors with prognostic value for intervention outcomes have been less studied [24, 30, 31]. This matched case-control study had two main objectives: 1) To compare children with ANSD and SNHL in open-set speech perception test scores through a matched case-control study design (1:2 ratio), and 2) To identify factors that show predictive value for post-CI speech perception outcomes. We hypothesized that with a matched case-control design, children with ANSD might achieve CI outcomes similar to peers with SNHL and that longer CI use and a lower age at CI activation might show prognostic value for optimal CI outcomes.

## Methods

### Participants

This retrospective case-control study was conducted at the Children’s Hospital of Eastern Ontario (CHEO) in Ottawa, Canada. Chart review and data extraction at CHEO was approved by the CHEO REB (Protocol No: 22/83X) with waived informed consent. All the data collected for this chart review were fully anonymized by the CHEO Research Group before granting access to the data. Additionally, the research received approval from the University of Ottawa Office of Research Ethics and Integrity (Ethics File Number # H-11-22-8149). The records of all children diagnosed with ANSD (n = 22), with CI outcome measures reported, between 01/09/2000 and 31/08/2022 were included in this study. Two research assistants, who were independent of the authors, conducted the data extraction. They were not privy to any information that could identify individual participants either during or after the data collection process. All children with ANSD included in this study exhibited bilateral auditory neuropathy, with no reported evidence of cochlear nerve deficiency (CND), as observed in MRI scans. For each child with ANSD, two children with SNHL with no evidence of cochleovestibular abnormalities were matched on biological sex, age of birth (months), age at CI activation, and length of using CIs. The ANSD diagnosis was based on inconsistency in audiologic findings in the clinical setting, consisting of present OAEs and/or CMs, absent or severely abnormal ABR, and various degrees of SNHL irrespective of present OAEs and/or CMs [32]. Children with less than six months of use of CIs were not included in the study. For data extraction, both electronic and paper records were reviewed by two research assistants independent of the authors. The list of data extracted for this study was: age of birth, HL characteristics (i.e., degree of HL and age of HL diagnosis), age at HA fitting, CI characteristics (i.e., type of CIs, the use of one or two CIs, and age at CI activation), medical conditions (additional disabilities [ADs], neonatal intensive care unit [NICU] history, and preterm birth [PB]), results of open-set speech perception test scores (i.e., outcome measures), and the length of follow-up with CIs.

### Outcome measures

The outcome measures in this study consisted of tests of open-set speech recognition, including the Phonetically Balanced Kindergarten (PBK) test utilizing both monosyllabic word (PBK-w) and phoneme (PBK-p) speech materials, as well as the Hearing in Noise Test (HINT) for children employing sentence materials in both quiet and noise conditions. For the PBK test, recorded speech materials comprising 25 monosyllabic words (PBK-w) and 80 phonemes (PBK-p) were presented at 60 dB SPL [33]. The HINT test sentences were administered in both quiet and noise conditions with speech-shaped noise at 10- and 5-dB signal-to-noise ratios (SNRs: HINT-10dB and HINT-5dB, respectively) at 60 dB SPL [34]. All speech perception tests were conducted within a sound booth in the clinical setting. In the chart review, the length of follow-up with CIs was defined based on the most recent follow-up session with outcome measures reported. Therefore, this measure referred to the difference between the date of CI activation and the date of speech test administration.

### Statistical analysis

Statistical analysis was conducted using SPSS Statistics 26.0 at a significance level of 0.05 or better. The univariate analysis of variance test was utilized to compare the two groups in speech perception outcomes. The Spearman’s rank correlation coefficient test was employed to construct the Correlation Matrix. A forward linear multiple regression model was performed to ascertain the variables that had predictive value for post-CI speech perception outcomes.

## Results

The present retrospective study reports the results of 66 children with CIs including 22 with ANSD (13 [59.10%] males and 9 [40.90%] females) and 44 children with SNHL (26 [59.10%] males and 18 [40.90%] females) aged 5 to 18 years old. Table 1 demonstrates demographic information for the two study groups including average age of birth (months), age at HL diagnosis, age at HA fitting, age at CI activation, biological sex, the use of one or two CIs, ADs/MCs (such as developmental delay, attention deficit hyperactivity disorder, autism, asthma, dyslexia, visual impairment, cerebral palsy, and cytomegalovirus), and the severity of HL. The two groups were not significantly different in age at HL diagnosis (p = 0.645), age at HA fitting (p = 0.953), age at CI activation (p = 0.484), and length of follow-up period (p = 0.389) (Table 1).

**Table 1:** Demographic characteristics of participants.

Demographic data	SNHL		ANSD	
	n	Mean (SD)	n	Mean (SD)
Age (months)	44	146.08 (48.97)	22	154 (43.16)
Age at HL diagnosis (months)	44	17.02 (7.91)	22	20.03 (32.64)
Age at HA Fitting (months)	44	25.43 (30.460	22	26.01 (32.90)
Age at CI activation (months)	44	47.78 (51.27)	22	50.40 (46.58)
Biological sex				
Male	26 (59.10)		13 (59.10)	
Female	18 (40.90)		9 (40.90)	
Length of follow-up period (months)	44 (100)	96.58 (54.39)	22 (100)	104.60 (60.66)
Severity of HL at outcome^a^				
Profound (> 80 dB)	44 (100)		18 (81.8)	
Severe (61–80 dB)			2 (9.10)	
Moderate (41–60 dB)			2 (9.10)	
Device				
Unilateral CI	15 (34.10)		8 (36.36)	
Bilateral CI	29 (65.90)		14 (64.64)	
Additional disabilities				
None	24 (54.54)		9 (40.90)	
≥1	20 (45.45)		13 (59.10)	
NICU admission	12 (27.27)		8 (36.36)	
Preterm birth	11 (25.0)		10 (45.45)	

^a^Averaged thresholds at 0.5, 1, 2, and 4 kHz in the better ear, including missing data for 3 children in the SNHL group and 2 in the ANSD group. ANSD, auditory neuropathy spectrum disorder; CI, cochlear implant; HA, hearing aid, HL, hearing loss; SD, standard deviation; SNHL, sensorineural hearing loss.

### Open-set speech perception test results

**Fig 1:**
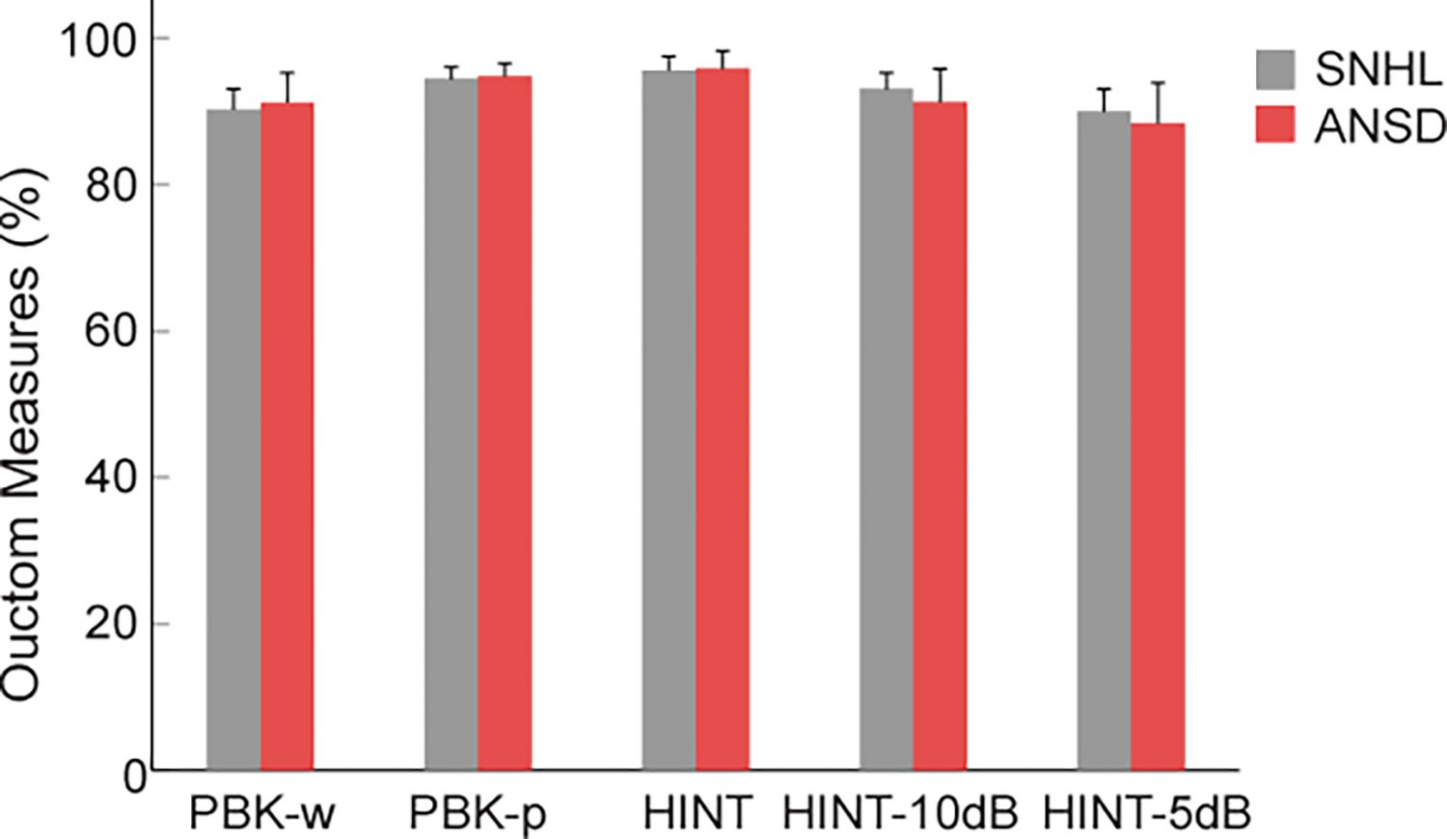
The average test scores in five speech perception outcome measures in the two study groups, including Phonetically Balanced Kindergarten with words (PBK-w) and phonemes (PBK-p) and Hearing in Noise Test in quiet (HINT) at two signal-to-noise ratios, HINT-5dB and HINT-10dB. No significant difference was found between the two groups. The bars exhibit mean ± 2SEM (standard error of the mean). ANSD, auditory neuropathy spectrum disorders; SNHL, sensorineural hearing loss.

No significant difference was observed between the SNHL and ANSD groups in the results of outcome measures (Fig 1), consisting of PBK-w test (F = 0.126, p = 0.724, partial η^2^ = 0.002, power = 0.064), PBK-p test (F = 0.054, p = 0.817, partial η^2^ = 0.001, power = 0.056), HINT test (F = 0.018, p = 0.892, partial η^2^ = 0.001, power = 0.052), HINT-10dB test (F = 0.298, p = 0.588, partial η^2^ = 0.012, power = 0.131), and HINT-5dB test (F = 0.054, p = 0.817, partial η^2^ = 0.006, power = 0.083).

### Correlation matrix

**Fig 2:**
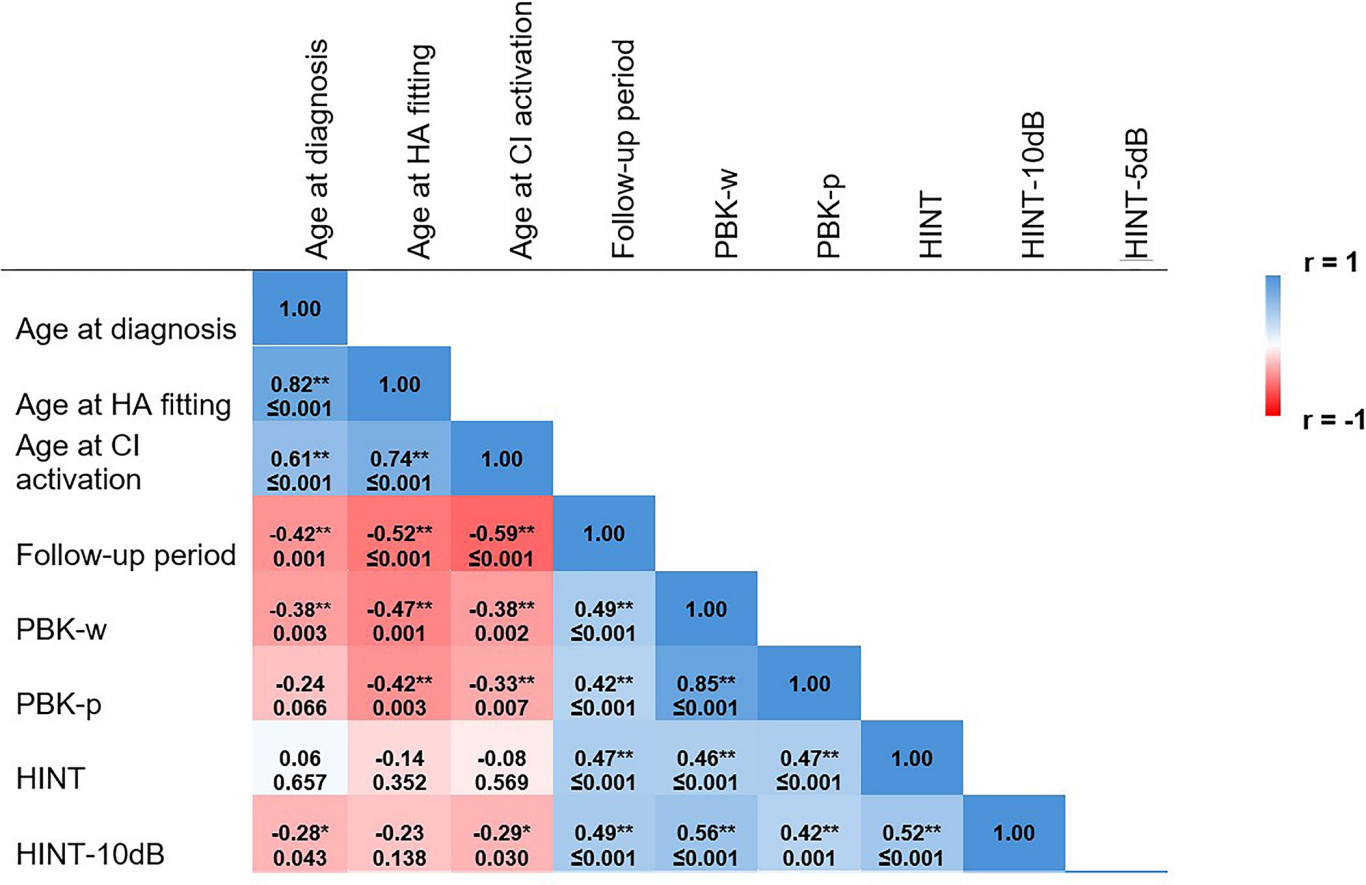
The correlation matrix highlights the strength of correlations between three critical ages and the results of five outcome measures. Each block shows the correlation between two variables and the associated p-value. Darker blue and red colors refer to greater positive and negative associations, respectively. HINT, Hearing in Noise Test (HINT-5dB and HINT-10dB); PBK-w/p, Phonetically Balanced Kindergarten with words or phonemes.

Fig 2 displays the Correlation Matrix, illustrating the strength of Spearman correlations between three critical ages (age of HL diagnosis, HA fitting, and CI activation) and the results of five open-set speech perception test scores (outcome measures). Each block represents the correlation between two variables and the associated p-value. Darker shades of blue and red indicate stronger positive and negative correlations, respectively.

### Predictors of intervention outcomes

A Forward Linear Multiple Regression Model was applied to determine factors that might have predictive value for the post-CI outcomes. The variables included in the model were age at HL diagnosis, age at HA fitting, age at CI activation, the length of follow-up with CIs, the use of one or two CIs, history of NICU admission and PB, presence of ADs, and biological sex. Table 2 summarizes the results of statistical analyses, including the regression coefficient (R square), F and p values, and standardized coefficient Beta. For example, among the variables included in the regression model, “the length of follow-up with CIs” was the only factor that showed a significant contribution (r = 0.328, p ≤ 0.001) to the PBK-w test results. Based on the regression coefficient, it could be interpreted that 32.80% of the outcome (i.e., the PBK-W test score) was explained by the length of follow-up with CIs. In addition, the positive magnitude of the beta value (0.531) indicated improved speech perception performance with increased length of CI use. The same results were obtained for the PBK-p test, corroborating the predictive value of the length of CI use for post-CI speech perception outcomes. For the HINT in quiet and HINT-10dB tests, the model identified two predictors of the outcomes. In addition to the length of follow-up with CIs, adding the “age at CI activation” significantly improved the predictive value of the model. However, for the HINT-5dB test, “bilateral amplification” was the factor that showed significant prognostic value.

**Table 2:** Using a Forward Linear Regression Model to identify factors that show predictive value for cochlear implant outcomes.

Speech perception test scores	Predictor	Regression coefficient (R-Square)	F	p	Standardized coefficient Beta
PBK-W	Follow-up period	0.328	21.578	≤0.001	0.531
PBK-P	Follow-up period	0.225	16.261	≤0.001	0.474
HINT in quiet	1. Follow-up period 2. Follow-up period and Age at CIs	0.256 0.316	16.618 10.998	≤0.001 ≤0.001	0.505 0.561
HINT– 10dB SNR	1. Follow-up period 2. Follow-up period and Age at CIs	0.225 0.319	13.643 10.758	0.001 ≤0.001	0.478 0.565
HINT– 5dB SNR	Bilateral amplification	0.212	7.664	0.013	0.399

CI: cochlear implant, HINT: Hearing in Noise Test in quiet and with two signal-to-noise ratios (SNR) of 10dB and 5dB, PBK: Phonetically Balanced Kindergarten test with word (PBK-W) and phoneme (PBK-P) speech materials.

## Discussion

The present study had three primary findings: 1) The ANSD and SNHL groups achieved high scores in post-CI speech perception outcomes, with no significant difference observed between the two groups. 2) Speech perception test scores were positively associated with a lower age at HA fitting and at CI activation and a longer follow-up with CIs. 3) Three variables including a longer use of CIs, age at CI activation, and the use of two CIs showed a predictive value for better speech perception outcomes. In the following paragraphs, the main findings are discussed.

### Post-CI open-set speech perception outcomes

In our study, both the ANSD and SNHL groups achieved optimal CI outcomes across five open-set speech perception test scores, in both quiet (PBK-w, PBK-p, and HINT in quiet) and noise (HINT-5dB and HINT-10dB) conditions, with no notable difference between the two groups. Children with ANSD displayed no indications of CND in MRI scan reports, suggesting a potential involvement of presynaptic mechanisms in ANSD. This finding is consistent with past studies indicating that the majority of children with presynaptic ANSD can attain speech understanding, language development, and communication outcomes equivalent to their peers with SNHL [7, 28]. However, children with postsynaptic ANSD, comprising almost 30% of children with ANSD [13], exhibit limited benefits from CIs and cannot develop functionally useful auditory communication skills [26, 27]. Thus, in ANSD, the lesion site along the auditory pathway carries prognostic significance, in which individuals with presynaptic ANSD or distal auditory nerve lesions (such as the involvement of inner hair cells or cochlear synapses) achieve optimal outcomes compared to children with postsynaptic involvement [3, 7, 22].

### Correlation matrix findings

As presented in the correlation matrix, a mild to high association was found between the results of speech perception test scores, with a lower magnitude of correlations between HINT-5dB with speech tests conducted in quiet. This finding is aligned with current evidence indicating speech perception in noise, especially at lower SNRs, as the most challenging listening condition for individuals with hearing loss [35], including children with cochlear implants [36–38]. Hearing-impaired individuals are highly noise intolerant, and despite remarkable improvements in current HA and CI technology, existing devices may not be very effective at combating background noise [39].

In the present study, open-set speech perception test scores were negatively associated with longer ages at HL diagnosis, HA fitting, and CI activation and positively associated with the length of follow-up with CIs (i.e., longer use of CIs). Our findings are aligned with past evidence regarding the impact of auditory deprivation and longer durations of HA or CI usage on spoken language development. For example, in two studies [40, 41], children with ANSD who received CIs before 24 months of age achieved higher scores in the Categories of Auditory Perception (CAP) test and Speech Intelligibility Rating (SIR) test compared to children who were implanted after 24 months of age. In a systematic review with a narrative synthesis of evidence [42] on implanted children with follow-up periods ranging from 6 months to 9 years, cochlear implantation before 24 months was found to be beneficial based on the scores of PBK and consonant-nucleus-consonant (CNC) tests. In addition, implantation before 12 months was associated with better speech production (using Diagnostic Evaluation of Articulation and Phonology and Infant-Toddler Meaningful Auditory Integration Scale [IT-MAIS]), auditory performance (the CAP-II score), and receptive language scores (based on the Preschool Language Scale combined with oral and written language skills and Peabody Picture Vocabulary Test). In another similar review, language outcomes for children implanted after 12 months decreased with the increased age of implantation [43]. In a study investigating cortical maturation, measured by P1 cortical auditory evoked potential (CAEP) latency, P1 CAEP latency was significantly correlated with children’s scores on the IT-MAIS. P1 CAEP responses were present in all children after implantation [44], compared to previous studies suggesting that only 50 [45] to 75 [46] of ANSD children with HAs showed CAEP responses. It was concluded that children fitted with CIs under two years of age were more likely to show age appropriate CAEP responses within six months after implantation, suggesting a possible sensitive period for cortical auditory development in ANSD [44].

The importance of early HL diagnosis and early hearing intervention is associated with the fact that the brain has high synaptic plasticity during childhood that progressively declines with age. This decline results from developmental mechanisms such as attenuated synaptic conductivity and the maturation of inhibitory neurons [47]. This age-related neural development shapes sensory object discrimination [48]. In addition, brain development is highly modulated by sensory inputs and profoundly reshaped by the lack of one sensory modality [49, 50]. According to MRI studies, early auditory deprivation leads to reduced white matter volume and integrity in the primary and secondary auditory cortex and spoken-language brain areas [51, 52]. The extent of structural neuroplasticity is an index of poor speech-language performance in late CI recipients [53]. In addition, auditory processing deficits due to functional intra-modal changes (e.g., the reduction of tonotopy, dynamic range, temporal resolution, and sensitivity to binaural cues) occur, which severely degrade the acuity of the auditory signal perceived [48, 54]. The findings of studies reviewed here underscore the crucial role of the sensitive period of auditory and spoken language development; a time limit of below 4 years, especially within the first two years of life [55–57], in which the central auditory system is highly plastic, and CI surgery could result in optimal outcomes [48].

### Factors with prognostic value for speech perception outcomes

We used a forward multiple regression model to determine the factors showing significant prognostic value for post-intervention outcomes. Among the variables included in the regression model, a longer follow-up with CIs, a lower age at CI activation, and the use of two CIs were the three factors that showed predictive value for optimal speech perception outcomes. Limited studies have reported the use of statistical models in identifying predictors of post-CI outcomes. In a prospective study by Ching et al. (2013) on 451 children in Australia with HL (30% with CIs, 10% with ANSD), age at CI activation, absence of ADs, higher maternal education, and female sex were reported as predictors for post-CI outcomes [24]. In a subsequent report by the same research group on language outcomes [58], the benefit of early intervention for language development increased as hearing loss increased. Children who received amplification at age 24 months had lower language scores than those fitted at 3 months, and children who received CIs at 24 months had lower language scores than those implanted at 6 months. In a retrospective study by le Roux et al. (2016) on 301 children with CIs (3.5% with ANSD) from five CI programs in South Africa, using two CIs, ADs/MCs, mainstream education, and ethnicities other than Caucasian contributed to post-CI outcomes [30].

An interesting finding in the present study was the significant contribution of the length of follow-up with CIs to the PBK (word and phoneme) and HINT in quiet and SNR 10dB test scores and the use of two CIs to HINT-5dB test scores. These findings underscore the crucial role of binaural hearing in speech perception, particularly in noisy environments. Even individuals with normal hearing find understanding speech in noise challenging [59]. Binaural hearing can substantially improve speech perception in noisy conditions and has been the focus of several trials for users of hearing technologies [60–62]. The binaural neural processing of the interaural level difference (ILD) and interaural time difference (ITD) can improve the internal SNR and consequently speech intelligibility [59, 63].

This study had two major strengths compared to past publications in the field. Firstly, employing a matched case-control study design on several factors contributing to CI outcomes allowed us to conduct a more precise comparison between children with ANSD and peers with SNHL. By matching the two groups, we not only observed similar progress and achievement of optimal CI outcomes in children with ANSD compared to the control group but also noted that ANSD was not identified as a predictor of the outcomes in the Forward Regression Model. This indicates similar speech perception progress with CIs in ANSD and SNHL under matched conditions. Secondly, this study was one of a few research efforts with a proper design reporting the factors with predictive value for optimal CI outcomes. The study’s limitation was a lack of information about other potential contributors, such as electrophysiological findings [31], maternal education, mode of communication at home, and socioeconomic status, which could affect the outcomes and their potential impact should be taken into account [24, 30, 58, 64]. Furthermore, the dataset used for this retrospective study did not encompass electrophysiological (e.g., electric auditory evoked potentials [eABR]) and genetic findings, which could offer insights into potential etiologies and enhance conclusions.

## Conclusions

In this retrospective matched (1:2) case-control study, we investigated post-CI speech perception outcomes in children with ANSD compared to peers with SNHL. Our findings revealed that under matched conditions, children with ANSD (without indications of postsynaptic involvement in MRI) achieved CI outcomes comparable to children with SNHL. This study contributes to the limited body of literature identifying factors predictive of post-CI outcomes. Utilizing a regression model, we identified three significant predictors of speech perception outcomes: duration of CI use, age at CI activation, and the utilization of bilateral CIs. Future research should consider additional potential contributors, such as genetics, socioeconomic status, and cognitive factors, to develop a more comprehensive model elucidating the determinants of post-CI speech perception outcomes.

## Supporting information

S1 File (PDF)

S2 File (PDF)

## References

[1] Bielecki I, Horbulewicz A, Wolan T. Prevalence and risk factors for auditory neuropathy spectrum disorder in a screened newborn population at risk for hearing loss. International Journal of Pediatric Otorhinolaryngology. 2012;76(11):1668–70. Epub 2012/09/04. doi: 10.1016/j.ijporl.2012.08.001 . 22939890

[2] Mittal R, Ramesh AV, Panwar SS, Nilkanthan A, Nair S, Mehra PR. Auditory neuropathy spectrum disorder: its prevalence and audiological characteristics in an Indian tertiary care hospital. International Journal of Pediatric Otorhinolaryngology. 2012;76(9):1351–4. Epub 2012/07/17. doi: 10.1016/j.ijporl.2012.06.005 . 22795739

[3] Shearer AE, Hansen MR. Auditory synaptopathy, auditory neuropathy, and cochlear implantation. Laryngoscope Investigative tolaryngology. 2019;4(4):429–40. Epub 2019/08/28. doi: 10.1002/lio2.288 ; PubMed Central PMCID: PMC6703118. 31453354 PMC6703118

[4] Rance G, Starr A. Pathophysiological mechanisms and functional hearing consequences of auditory neuropathy. Brain. 2015;138(Pt 11):3141–58. Epub 2015/10/16. doi: 10.1093/brain/awv270 . 26463676

[5] Rance G, Starr A. Auditory neuropathy/dys-synchrony. In: Seewald R, Tharpe A, editors. Comprehensive handbook of pediatric audiology San Diego: Plural Publishing; 2011. p. 225–42.

[6] Starr A, Rance G. Auditory neuropathy. In: Celesia G, Hickok G, editors. Handbook of Clinical Neurology. 129. Edinburgh Elsevier; 2015. p. 495–508. 25726287 10.1016/B978-0-444-62630-1.00028-7

[7] Jafari Z, Fitzpatrick EM, Schramm DR, Rouillon I, Koravand A. An Umbrella Review of Cochlear Implant Outcomes in Children With Auditory Neuropathy. Journal of Speech, Language, and Hearing Research: JSLHR. 2023;66(10):4160–76. Epub 2023/08/30. doi: 10.1044/2023_jslhr-23-00128 . 37647160

[8] Rance G. Auditory neuropathy/dys-synchrony and its perceptual consequences. Trends in Amplification. 2005;9(1):1–43. Epub 2005/05/28. doi: 10.1177/108471380500900102 ; PubMed Central PMCID: PMC4111505. 15920648 PMC4111505

[9] Hood LJ. Auditory Neuropathy/Auditory Synaptopathy. Otolaryngologic Clinics of North America. 2021;54(6):1093–100. Epub 2021/09/19. doi: 10.1016/j.otc.2021.07.004 . 34535280

[10] Jafari Z, Malayeri S, Ashayeri H, Farahani MA. Adults with auditory neuropathy: comparison of auditory steady-state response and pure-tone audiometry. Journal of the American Academy of Audiology. 2009;20(10):621–8. Epub 2010/05/28. doi: 10.3766/jaaa.20.10.4 . 20503800

[11] Manchaiah VK, Zhao F, Danesh AA, Duprey R. The genetic basis of auditory neuropathy spectrum disorder (ANSD). International Journal of Pediatric Otorhinolaryngology. 2011;75(2):151–8. Epub 2010/12/24. doi: 10.1016/j.ijporl.2010.11.023 . 21176974

[12] James AL, Osborn HA, Osman H, Papaioannou V, Gordon KA. The limitation of risk factors as a means of prognostication in auditory neuropathy spectrum disorder of perinatal onset. International Journal of Pediatric Otorhinolaryngology. 2020;135:110112. Epub 2020/06/06. doi: 10.1016/j.ijporl.2020.110112 . 32502912

[13] Hall J. eHandbook of Auditory Evoked Responses: Principles, Procedures & Protocols: Pearson Education, Inc.; 2015.

[14] Huang Y, Yang J, Duan M. Auditory neuropathy: from etiology to management. Current Opinion in Otolaryngology & Head and Neck Surgery. 2022;30(5):332–8. Epub 2022/08/09. doi: 10.1097/MOO.0000000000000829 . 35939320

[15] Saidia AR, Ruel J, Bahloul A, Chaix B, Venail F, Wang J. Current Advances in Gene Therapies of Genetic Auditory Neuropathy Spectrum Disorder. Journal of Clinical Medicine. 2023;12(3). Epub 2023/02/12. doi: 10.3390/jcm12030738 ; PubMed Central PMCID: PMC9918155. 36769387 PMC9918155

[16] Wolfe J. Cochlear Implants: Audiologic Management and Considerations for Implantable Hearing Devices. San Diego: Plural Publishing Inc.; 2020.

[17] Myers K, Nicholson N. Cochlear Implant Behavioral Outcomes for Children With Auditory Neuropathy Spectrum Disorder: A Mini-Systematic Review. American Journal of Audiology. 2021;30(3):777–89. Epub 2021/07/24. doi: 10.1044/2021_AJA-20-00175 . 34297601

[18] Walker E, McCreery R, Spratford M, Roush P. Children with Auditory Neuropathy Spectrum Disorder Fitted with Hearing Aids Applying the American Academy of Audiology Pediatric Amplification Guideline: Current Practice and Outcomes. Journal of the American Academy of Audiology. 2016;27(3):204–18. Epub 2016/03/12. doi: 10.3766/jaaa.15050 ; PubMed Central PMCID: PMC4789798. 26967362 PMC4789798

[19] Berlin CI, Hood LJ, Morlet T, Wilensky D, Li L, Mattingly KR, et al. Multi-site diagnosis and management of 260 patients with auditory neuropathy/dys-synchrony (auditory neuropathy spectrum disorder). International Journal of Audiology. 2010;49(1):30–43. Epub 2010/01/08. doi: 10.3109/14992020903160892 . 20053155

[20] Ching TY, Day J, Dillon H, Gardner-Berry K, Hou S, Seeto M, et al. Impact of the presence of auditory neuropathy spectrum disorder (ANSD) on outcomes of children at three years of age. International Journal of Audiology. 2013;52 Suppl 2(0 2):S55–64. Epub 2013/12/20. doi: 10.3109/14992027.2013.796532 ; PubMed Central PMCID: PMC3869001. 24350696 PMC3869001

[21] Kuchta J. Twenty-five years of auditory brainstem implants: perspectives. Acta Neurochirurgica Supplement. 2007;97(Pt 2):443–9. Epub 2007/08/19. doi: 10.1007/978-3-211-33081-4_51 . 17691334

[22] Chaudhry D, Chaudhry A, Muzaffar J, Monksfield P, Bance M. Cochlear Implantation Outcomes in Post Synaptic Auditory Neuropathies: A Systematic Review and Narrative Synthesis. The Journal of International Advanced Otology. 2020;16(3):411–31. Epub 2020/11/03. doi: 10.5152/iao.2020.9035 ; PubMed Central PMCID: PMC7901461. 33136025 PMC7901461

[23] Jafari Z, Kolb BE, Mohajerani MH. A systematic review of altered resting-state networks in early deafness and implications for cochlear implantation outcomes. European Journal of Neuroscience. 2024. Epub 20240305. doi: 10.1111/ejn.16295 . 38441248

[24] Ching TY, Dillon H, Marnane V, Hou S, Day J, Seeto M, et al. Outcomes of early- and late-identified children at 3 years of age: findings from a prospective population-based study. Ear and Hearing. 2013;34(5):535–52. Epub 2013/03/07. doi: 10.1097/AUD.0b013e3182857718 ; PubMed Central PMCID: PMC3681915. 23462376 PMC3681915

[25] Niparko JK, Tobey EA, Thal DJ, Eisenberg LS, Wang NY, Quittner AL, et al. Spoken language development in children following cochlear implantation. JAMA. 2010;303(15):1498–506. Epub 2010/04/22. doi: 10.1001/jama.2010.451 ; PubMed Central PMCID: PMC3073449. 20407059 PMC3073449

[26] Peng KA, Kuan EC, Hagan S, Wilkinson EP, Miller ME. Cochlear Nerve Aplasia and Hypoplasia: Predictors of Cochlear Implant Success. Otolaryngology-Head and Neck Surgery. 2017;157(3):392–400. Epub 2017/07/05. doi: 10.1177/0194599817718798 . 28675079

[27] Vesseur A, Free R, Snels C, Dekker F, Mylanus E, Verbist B, et al. Hearing Restoration in Cochlear Nerve Deficiency: the Choice Between Cochlear Implant or Auditory Brainstem Implant, a Meta-analysis. Otology & Neurotology. 2018;39(4):428–37. Epub 2018/03/02. doi: 10.1097/MAO.0000000000001727 . 29494474

[28] Bo D, Huang Y, Wang B, Lu P, Chen WX, Xu ZM. Auditory and Speech Outcomes of Cochlear Implantation in Children With Auditory Neuropathy Spectrum Disorder: A Systematic Review and Meta-Analysis. The Annals of Otology, Rhinology, and Laryngology. 2022:34894221092201. Epub 2022/05/03. doi: 10.1177/00034894221092201 . 35499129

[29] Jeddi Z, Jafari Z, Motasaddi Zarandy M, Kassani A. Aural rehabilitation in children with cochlear implants: a study of cognition, social communication, and motor skill development. Cochlear Implants International. 2014;15(2):93–100. Epub 20140103. doi: 10.1179/1754762813Y.0000000060 . 24597636

[30] le Roux T, Vinck B, Butler I, Cass N, Louw L, Nauta L, et al. Predictors of pediatric cochlear implantation outcomes in South Africa. International Journal of Pediatric Otorhinolaryngology. 2016;84:61–70. Epub 2016/04/12. doi: 10.1016/j.ijporl.2016.02.025 . 27063755

[31] Jafari J, Fitzpatrick EM, Schramm DR, Rouillon R, Koravand A. Prognostic Value of Electrophysiologic and MRI Findings for Pediatric Cochlear Implant Outcomes: A Systematic Review. American Journal of Audiology. 2024. 10.1044/2024_AJA-23-00272 39018270

[32] Hayes D, Sininger Y. Guidelines for Identification and Management of Infants and Young Children with Auditory Neuropathy Spectrum Disorder. NHS 2008, Como, Italy2008.

[33] He S, Grose JH, Teagle HF, Woodard J, Park LR, Hatch DR, et al. Acoustically evoked auditory change complex in children with auditory neuropathy spectrum disorder: a potential objective tool for identifying cochlear implant candidates. Ear and Hearing. 2015;36(3):289–301. Epub 2014/11/26. doi: 10.1097/AUD.0000000000000119 ; PubMed Central PMCID: PMC4409935. 25422994 PMC4409935

[34] Eisenberg LS, Fisher LM, Johnson KC, Ganguly DH, Grace T, Niparko JK. Sentence Recognition in Quiet and Noise by Pediatric Cochlear Implant Users: Relationships to Spoken Language. Otology & Neurotology. 2016;37(2):e75–81. Epub 2016/01/13. doi: 10.1097/MAO.0000000000000910 ; PubMed Central PMCID: PMC4712714. 26756159 PMC4712714

[35] Helfer KS, Wilber LA. Hearing loss, aging, and speech perception in reverberation and noise. Journal of Speech and Hearing Research. 1990;33(1):149–55. Epub 1990/03/01. doi: 10.1044/jshr.3301.149 . 2314073

[36] Rance G, Barker E, Mok M, Dowell R, Rincon A, Garratt R. Speech perception in noise for children with auditory neuropathy/dys-synchrony type hearing loss. Ear and Hearing. 2007;28(3):351–60. Epub 2007/05/09. doi: 10.1097/AUD.0b013e3180479404 . 17485984

[37] Ma C, Fried J, Nguyen SA, Schvartz-Leyzac KC, Camposeo EL, Meyer TA, et al. Longitudinal Speech Recognition Changes After Cochlear Implant: Systematic Review and Meta-analysis. The Laryngoscope. 2023;133(5):1014–24. Epub 2022/08/26. doi: 10.1002/lary.30354 . 36004817

[38] Dorman MF, Gifford RH. Speech Understanding in Complex Listening Environments by Listeners Fit With Cochlear Implants. Journal of Speech, Language, and Hearing Research: JSLHR. 2017;60(10):3019–26. Epub 2017/10/20. doi: 10.1044/2017_JSLHR-H-17-0035 ; PubMed Central PMCID: PMC5945071. 29049602 PMC5945071

[39] Healy EW, Yoho SE. Difficulty understanding speech in noise by the hearing impaired: underlying causes and technological solutions. Annual International Conference of the IEEE Engineering in Medicine and Biology Society IEEE Engineering in Medicine and Biology Society Annual International Conference. 2016;2016:89–92. Epub 2017/03/09. doi: 10.1109/EMBC.2016.7590647 . 28268288

[40] Liu Y, Dong R, Li Y, Xu T, Li Y, Chen X, et al. Effect of age at cochlear implantation on auditory and speech development of children with auditory neuropathy spectrum disorder. Auris, Nasus, Larynx. 2014;41(6):502–6. Epub 2014/09/10. doi: 10.1016/j.anl.2014.06.001 . 25194855

[41] Daneshi A, Mirsalehi M, Hashemi SB, Ajalloueyan M, Rajati M, Ghasemi MM, et al. Cochlear implantation in children with auditory neuropathy spectrum disorder: A multicenter study on auditory performance and speech production outcomes. International Journal of Pediatric Otorhinolaryngology. 2018;108:12–6. Epub 2018/04/02. doi: 10.1016/j.ijporl.2018.02.004 . 29605339

[42] Bruijnzeel H, Ziylan F, Stegeman I, Topsakal V, Grolman W. A Systematic Review to Define the Speech and Language Benefit of Early (<12 Months) Pediatric Cochlear Implantation. Audiology & Neuro-otology. 2016;21(2):113–26. Epub 2016/04/14. doi: 10.1159/000443363 . 27074000

[43] Ruben RJ. Language development in the pediatric cochlear implant patient. Laryngoscope Investigative Otolaryngology. 2018;3(3):209–13. Epub 2018/08/01. doi: 10.1002/lio2.156 ; PubMed Central PMCID: PMC6057214. 30062136 PMC6057214

[44] Cardon G, Sharma A. Central auditory maturation and behavioral outcome in children with auditory neuropathy spectrum disorder who use cochlear implants. International Journal of Audiology. 2013;52(9):577–86. Epub 2013/07/04. doi: 10.3109/14992027.2013.799786 ; PubMed Central PMCID: PMC3781925. 23819618 PMC3781925

[45] Rance G, Cone-Wesson B, Wunderlich J, Dowell R. Speech perception and cortical event-related potentials in children with auditory neuropathy. Ear and Hearing. 2002;23(3):239–53. Epub 2002/06/20. doi: 10.1097/00003446-200206000-00008 . 12072616

[46] Sharma A, Cardon G, Henion K, Roland P. Cortical maturation and behavioral outcomes in children with auditory neuropathy spectrum disorder. International Journal of Audiology. 2011;50(2):98–106. Epub 2011/01/27. doi: 10.3109/14992027.2010.542492 ; PubMed Central PMCID: PMC3735347. 21265637 PMC3735347

[47] Kolb B, Muhammad A. Harnessing the power of neuroplasticity for intervention. Frontiers in Human Neuroscience. 2014;8:377. Epub 2014/07/16. doi: 10.3389/fnhum.2014.00377 ; PubMed Central PMCID: PMC4072970. 25018713 PMC4072970

[48] Kral A, Dorman MF, Wilson BS. Neuronal Development of Hearing and Language: Cochlear Implants and Critical Periods. Annual Review of Neuroscience. 2019;42:47–65. Epub 2019/01/31. doi: 10.1146/annurev-neuro-080317-061513 . 30699049

[49] Bavelier D, Neville HJ. Cross-modal plasticity: where and how? Nature Reviews Neuroscience. 2002;3(6):443–52. Epub 2002/06/04. doi: 10.1038/nrn848 . 12042879

[50] Merabet LB, Pascual-Leone A. Neural reorganization following sensory loss: the opportunity of change. Nature Reviews Neuroscience. 2010;11(1):44–52. Epub 2009/11/26. doi: 10.1038/nrn2758 ; PubMed Central PMCID: PMC3898172. 19935836 PMC3898172

[51] Hribar M, Suput D, Carvalho AA, Battelino S, Vovk A. Structural alterations of brain grey and white matter in early deaf adults. Hearing Research. 2014;318:1–10. Epub 2014/09/30. doi: 10.1016/j.heares.2014.09.008 . 25262621

[52] Karns CM, Stevens C, Dow MW, Schorr EM, Neville HJ. Atypical white-matter microstructure in congenitally deaf adults: A region of interest and tractography study using diffusion-tensor imaging. Hearing Research. 2017;343:72–82. Epub 2016/07/31. doi: 10.1016/j.heares.2016.07.008 . 27473505 PMC11668359

[53] Simon M, Campbell E, Genest F, MacLean MW, Champoux F, Lepore F. The Impact of Early Deafness on Brain Plasticity: A Systematic Review of the White and Gray Matter Changes. Frontiers in Neuroscience. 2020;14:206. Epub 2020/04/16. doi: 10.3389/fnins.2020.00206 ; PubMed Central PMCID: PMC7135892. 32292323 PMC7135892

[54] Kral A, Yusuf PA, Land R. Higher-order auditory areas in congenital deafness: Top-down interactions and corticocortical decoupling. Hearing Research. 2017;343:50–63. Epub 2016/09/18. doi: 10.1016/j.heares.2016.08.017 . 27637669

[55] Kral A, Sharma A. Developmental neuroplasticity after cochlear implantation. Trends in Neurosciences. 2012;35(2):111–22. Epub 2011/11/23. doi: 10.1016/j.tins.2011.09.004 ; PubMed Central PMCID: PMC3561718. 22104561 PMC3561718

[56] Sharma A, Campbell J. A sensitive period for cochlear implantation in deaf children. The Journal of Maternal-fetal & Neonatal Medicine. 2011;24 Suppl 1(0 1):151–3. Epub 2011/09/29. doi: 10.3109/14767058.2011.607614 ; PubMed Central PMCID: PMC3743531. 21942615 PMC3743531

[57] Shirvani S, Jafari Z, Motasaddi Zarandi M, Jalaie S, Mohagheghi H, Tale MR. Emotional Perception of Music in Children With Bimodal Fitting and Unilateral Cochlear Implant. The Annals of Otology, Rhinology, and Laryngology. 2016;125(6):470–7. Epub 2015/12/19. doi: 10.1177/0003489415619943 . 26681623

[58] Ching TYC, Dillon H, Button L, Seeto M, Van Buynder P, Marnane V, et al. Age at Intervention for Permanent Hearing Loss and 5-Year Language Outcomes. Pediatrics. 2017;140(3). Epub 2017/09/03. doi: 10.1542/peds.2016-4274 ; PubMed Central PMCID: PMC5574730 conflicts of interest to disclose. 28864712 PMC5574730

[59] Wagner L, Geiling L, Hauth C, Hocke T, Plontke S, Rahne T. Improved binaural speech reception thresholds through small symmetrical separation of speech and noise. PloS One. 2020;15(8):e0236469. Epub 2020/08/07. doi: 10.1371/journal.pone.0236469 ; PubMed Central PMCID: PMC7406049. 32756594 PMC7406049

[60] Williges B, Wesarg T, Jung L, Geven LI, Radeloff A, Jürgens T. Spatial Speech-in-Noise Performance in Bimodal and Single-Sided Deaf Cochlear Implant Users. Trends in Hearing. 2019;23:2331216519858311. Epub 2019/08/01. doi: 10.1177/2331216519858311 ; PubMed Central PMCID: PMC6669847. 31364496 PMC6669847

[61] Rader T, Fastl H, Baumann U. Speech perception with combined electric-acoustic stimulation and bilateral cochlear implants in a multisource noise field. Ear and Hearing. 2013;34(3):324–32. Epub 2012/12/25. doi: 10.1097/AUD.0b013e318272f189 . 23263408

[62] Kraaijenga VJ, van Zon A, Smulders YE, Ramakers GG, Van Zanten GA, Stokroos RJ, et al. Development of a Squelch Effect in Adult Patients After Simultaneous Bilateral Cochlear Implantation. Otology & Neurotology. 2016;37(9):1300–6. Epub 2016/09/01. doi: 10.1097/MAO.0000000000001185 . 27579836

[63] Lingner A, Grothe B, Wiegrebe L, Ewert SD. Binaural Glimpses at the Cocktail Party? Journal of the Association for Research in Otolaryngology: JARO. 2016;17(5):461–73. Epub 2016/07/15. doi: 10.1007/s10162-016-0575-7 ; PubMed Central PMCID: PMC5023537. 27412529 PMC5023537

[64] Jeddi Z, Jafari Z, Motasaddi Zarandy M. Effects of parents’ level of education and economic status on the age at cochlear implantation in children. Iranian Journal of Otorhinolaryngology. 2012;24(66):7–15. ; PubMed Central PMCID: PMC3846204. 24303378 PMC3846204

