# Supplementary material for: Predictors of cochlear implant outcomes in pediatric auditory neuropathy: A matched case-control study
Source: PLoS One. 2024 May 29;19(5):e0304316. doi: 10.1371/journal.pone.0304316 (PMC11135674; doi:10.1371/journal.pone.0304316)
Supplement: S1 File — (PDF) [file pone.0304316.s001.pdf]

Whittingham, JoAnne

From: nanderson@cheo.on.ca
Sent: Friday, October 28, 2022 9:22 AM
To: Lessard, Chantal
Cc: Sokalski, Ashley; Whittingham, JoAnne; Anderson, Natalie
Subject: REB Protocol No 22/83X - Final Approval - Delegated Review

EXTERNAL MAIL*

CHEO REB
Letter of Approval

REB Protocol No: 22/83X
ROMEO File No: 20221267
Principal Investigator: Dr. Chantal Lessard
Protocol Title: CHEOREB# 22/83X - Predictors of Amplification Outcomes in Children with Auditory Neuropathy Spectrum Disorders (ANSD)

Protocol Status: Active

Approval Date: October 28, 2022
Approval Expiry Date: October 15, 2023

The CHEO REB has conducted a delegated review and determined that the conditions of approval have been satisfied for the above-named study. Approval is valid for the period indicated above. This research study is to be conducted by the investigator noted above. Annual renewals or study closures must be completed before the expiry date noted above.

REB members involved in the study do not participate in the review, deliberations, or decision.

Documents Approved:

Document Name	Comments	Version Date
Case Report Form	Case report form (variable list)	2022/10/12
Protocol	Protocol Version 4	2022/10/12

Any modifications made to the study must be reviewed and approved by the REB prior to implementation, except when necessary to eliminate immediate danger or hazard(s) to study participants or when the change(s) involves administrative aspects of the study. Investigators must promptly alert the REB of any changes that increase the risk to participants or affect the safety of participants, all unanticipated and harmful events that occur, and new information that significantly impact the conduct of the study.

The CHEO REB operates in compliance with, and is constituted in accordance with, the requirements of the Tri-Council Policy Statement: Ethical Conduct of Research Involving Humans (TCPS 2); the International Conference on

Harmonization Good Clinical Practice Consolidated Guideline (ICH GCP); Part C, Division 5 of the Food and Drug Regulations; Part 4 of the Natural Health Products Regulations; and Part 3 of the Medical Devices Regulations and the provisions of the Ontario Personal Health Information Protection Act (PHIPA 2004) and its applicable regulations. The CHEO REB is registered with the U.S. Department of Health and Human Services (DHHS) Office for Human Research Protection (OHRP).

Please do not hesitate to contact the Research Ethics Office if you have any questions.

Best wishes for the successful conduct of your research.

Cécile Bensimon, MA, PhD
Chair, Research Ethics Board
Présidente, Comité d'éthique de la recherche

***EXTERNAL MAIL:** Caution, this email came to you from outside of CHEO. Do not click any links or open any attachments unless you know the sender and are certain the content is safe.
