# Supplementary material for: Predictors of cochlear implant outcomes in pediatric auditory neuropathy: A matched case-control study
Source: PLoS One. 2024 May 29;19(5):e0304316. doi: 10.1371/journal.pone.0304316 (PMC11135674; doi:10.1371/journal.pone.0304316)
Supplement: S2 File — (PDF) [file pone.0304316.s002.pdf]

Université d'Ottawa
Bureau d'éthique et d'intégrité de la recherche

University of Ottawa
Office of Research Ethics and Integrity

Lettre d’approbation administrative | Letter of administrative approval

**Numéro de dossier / Ethics File Number**	H-11-22-8149
**Titre du projet / Project Title**	Predictors of Amplification Outcomes in Children with Auditory Neuropathy Spectrum Disorders (ANSD)
**Type de projet / Project Type**	Recherche de professeur / Professor's research project
**CÉR primaire / Primary REB**	
**Statut du projet / Project Status**	Approuvé / Approved
**Date d'approbation (jj/mm/aaaa) / Approval Date (dd/mm/yyyy)**	18/11/2022
**Date d'expiration (jj/mm/aaaa) / Expiry Date (dd/mm/yyyy)**	16/10/2023

Équipe de recherche / Research Team

Chercheur / Researcher	Affiliation	Role
Amineh KORAVAND	École des sciences de la réadaptation / School of Rehabilitation Sciences	Chercheur Principal / Principal Investigator
Zahra JAFARI	École des sciences de la réadaptation / School of Rehabilitation Sciences	Co-chercheur principal / Co-principal investigator
Chantal LESSARD	CHEO	Co-chercheur / Co-investigator
Ashley SOKALSKI	CHEO	Coordonnateur de recherche / Research Coordinator
Jennifer Ti NGUYEN	École des sciences de la réadaptation / School of Rehabilitation Sciences	Assistant de recherche / Research Assistant

Conditions spéciales ou commentaires / Special conditions or comments:

CHEO REB Protocol No: 22/83X

550, rue Cumberland, pièce 154
Ottawa (Ontario) K1N 6N5 Canada
550 Cumberland Street, Room 154
Ottawa, Ontario K1N 6N5 Canada

613-562-5387 • 613-562-5338 • ethique@uOttawa.ca / ethics@uOttawa.ca
www.recherche.uottawa.ca/deontologie | www.recherche.uottawa.ca/ethics

Université d'Ottawa
Bureau d'éthique et d'intégrité de la recherche

University of Ottawa
Office of Research Ethics and Integrity

L'Université d'Ottawa a signé une Entente, conforme aux exigences de la plus récente version de l'EPTC et tout autre règlement ou législation applicable, permettant au CÉR ci-haut nommé d'être désigné comme CÉR primaire pour les projets de recherche où

1) les activités principales de recherche sont menées sous l'autorité ou sous les auspices de l'établissement lié au CÉR primaire et

2) Une partie du projet est également réalisé sous l'autorité ou sous les auspices de l'Université d'Ottawa.

Cette lettre confirme que l'Université d'Ottawa a autorisé que le CÉR primaire soit le CÉR officiel pour l'évaluation et la supervision de ce projet de recherche. Ceci n'est pas une approbation éthique.

Afin de nous aider à garder votre dossier à jour, veuillez soumettre une copie de toutes demandes de modification, renouvellement d'approbation éthique etc. soumis à et approuvé par le CÉR primaire dès qu'elles sont disponibles.

Cette approbation administrative est valide pour la durée indiquée ci-haut et est sujette aux conditions énumérées dans la section intitulée « Conditions spéciales ou commentaires ».

The University of Ottawa has signed an Agreement, compliant with current TCPS guidelines and any other applicable guidelines or legislation regarding multisite review, allowing the REB named above to serve as Board of Record (BoR) for research projects where

1) the main research activities are conducted within the auspices or jurisdiction of the BoR's institution and

2) parts of the project are also conducted under the jurisdiction or auspices of the University of Ottawa.

This letter confirms that the University of Ottawa has authorized the REB named above to serve as Board of Record for the review and oversight of this research project. This is not an REB approval.

In order to help us keep your file up to date, please submit a copy of all amendment requests, project renewals or any other changes submitted to and approved by the BoR, as they become available.

Administrative approval is valid for the period indicated above and is subject to the conditions listed in the section entitled «Special conditions or comments».

Catherine PAQUET
Directeur / Director
Pour/For **Daniel LAGAREC** Président(e) du/ Chair of the **Comité d'éthique de la recherche en sciences de la santé et sciences / Health Sciences and Sciences Research Ethics Board**

550, rue Cumberland, pièce 154
Ottawa (Ontario) K1N 6N5 Canada

550 Cumberland Street, Room 154
Ottawa, Ontario K1N 6N5 Canada

613-562-5387 • 613-562-5338 • ethique@uOttawa.ca / ethics@uOttawa.ca
www.recherche.uottawa.ca/deontologie | www.recherche.uottawa.ca/ethics
